# Regarding the Nature of the Blue Pigment in Arils of *Ravenala Madagascariensis* Sonn. (*Strelitziaceae*)

**DOI:** 10.1021/acsomega.4c11177

**Published:** 2025-03-26

**Authors:** Beatriz Paiva Nogueira, Marcos Miguel Quimas do Amaral, Arthur Girard Carpanez, Ari Sergio de Oliveira Lemos, Frederico Francisco Fernandes, Nádia Sílvia Somavilla, Marcone Augusto Leal de Oliveira, William de Castro Borges, Eveline Gomes Vasconcelos, Richard Michael Grazul, Priscila de Faria-Pinto

**Affiliations:** † Departamento de Bioquímica, Instituto de Ciências Biológicas, Universidade Federal de Juiz de Fora, 36036-900 Juiz de Fora, Minas Gerais, Brazil; ‡ Departamento de Botânica, Instituto de Ciências Biológicas, Universidade Federal de Juiz de Fora, 36036-900 Juiz de Fora, Minas Gerais, Brazil; § Departamento de Química, Instituto de Ciências Exatas, Universidade Federal de Juiz de Fora, 36036-900 Juiz de Fora, Minas Gerais, Brazil; ∥ Laboratório de Enzimologia e Proteômica, Universidade Federal de Ouro Preto, 35400-000 Ouro Preto, Minas Gerais, Brazil

## Abstract

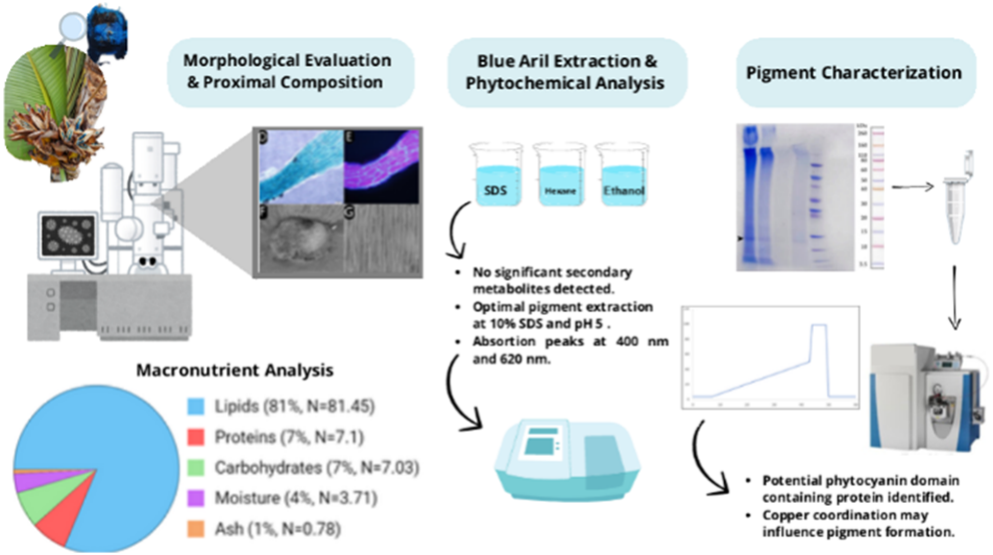

*Ravenala madagascariensis*, the Traveler’s tree, is native to Madagascar but has adapted globally and is utilized for medical and cosmetic purposes. Focusing on the brightly blue colored arils of the plant’s seeds, this study aimed to examine their morphological and physicochemical properties and to shed light upon the previously undescribed structure of the pigment. Plant arils were examined by energy-dispersive X-ray spectroscopy (EDS) and scanning electron microscopy (SEM). Centesimal analyses indicated a lipid-rich composition (81.45%), carbohydrates (7.03%), moisture (3.71%), ash (0.78%), and proteins/peptides (7.1%). Analysis of the fatty acid methyl esters obtained from the lipid fraction by GC–MS indicated a composition of 41% palmitic, 14% stearic, 34% oleic and 7% linoleic acids. A battery of qualitative tests employing hexane, ethanol, and 10% aqueous sodium dodecyl sulfate (SDS) extracts of *R. madagascariensis* arils for the presence the following secondary metabolites was performed: alkaloids, triterpenes and phytosteroids, coumarins, phenolics, flavonoids, anthroquiones, saponins and tannins. Results for all classes were negative or, in one case, inconclusive. Factorial optimization of extraction conditions for isolation of the pigment was performed indicating 10% aqueous SDS at pH = 5 to be the most efficient to remove the pigment from the arils, while spectrophotometer scans indicated absorption peaks at 400 and 620 nm. Thin layer chromatography (TLC) of a 6 M HCl digest of the arils tested positive for amino acids when developed with ninhydrin. SDS-PAGE electrophoresis revealed a 14 kDa protein profile and the peptide fractions obtained after partial degradation with trypsin were analyzed by (UHPLC/MS/MS), lending important structural information that strongly suggests the presence of a phytocyanin (Blue Copper Protein). Analysis of our data and by comparison to the NCBI BLAST data bank indicated a phytocyanin domain-containing protein, responsible for the blue coloration of Ravenala arils due to copper coordination with the protein.

## 1 Introduction

Synthetic and natural pigments have been used by humans for a long time in various fields, ranging from the food and pharmaceutical industries to textiles and the arts.^1^ These natural components, responsible for the origin of the tones and shades of color, can be obtained from fruits, leaves, flowers, bacteria, fungi, and insects. The colors that prevail in vegetables, for example, predominantly result from pigment categories such as chlorophyll, carotenoids, betalains and anthocyanins.^2^ Despite increasing consumer preference for the use of natural colorants, not all colors are easily obtained in nature, as is the case with blue dyes. Anthocyanins, responsible for blue and purple colors in fruits and vegetables, are the only commercially used natural sources of this hue but face stability limitations. Therefore, to achieve the blue color and other derived colors, it is often necessary to resort to synthetic blue dyes.^3^

*Ravenala madagascariensis*, popularly known as the “traveler’s tree,” is a plant belonging to the Strelitziaceae family, originating from the island of Madagascar but successfully adapting to diverse tropical regions where it is widely cultivated as an ornamental. Anthropological studies of native populations have revealed a wide variety of uses including: construction of dwellings (trunks), tools and utensils and as a food source (boiled trunk heart).^4,5^ The plant is also used phytotherapically against a plethora of ailments including: coughs, stomachache, urine retention, diabetes, diarrhea, edema, kidney stones and hypertension.^5,6^ Recent studies have shown antioxidant, antimicrobial, enzymatic inhibition, antidiabetic and cytotoxic effects in vitro and in vivo.^6−8^

The morphology of the group includes a woody stem, bracts, leathery leaves, and a structure surrounding the seeds called aril. *Ravenala* presents distinctive traits that set it apart from other genera in its family, such as a trunk resembling that of a palm tree, leaves reminiscent of banana trees but with a vertical orientation, and its seeds are enveloped by this aril with a blue color of unusual chemical stability, showing significant durability and maintaining pigmentation even after cell death.^9^ Arils are structures found around seeds that often exhibit vibrant colors. Their function is linked to pollination, attracting animals that consume the arils and thus disperse the seeds. In Madagascar, lemurs play an important role as pollinators, attracted by the colors, including blue, of the arils.^10^ However, the chemical nature of the pigmentation of these structures is not yet understood. To our knowledge, the first work with *Ravenala* was the treatise by Francisco Giral in 1946, a great part of whose work we have validated.^11^ Due to the paucity of data in the literature that describes the nature or origin of the coloring on the arils, the current study focuses on investigating: the morphology, the chemical composition, the best form of extraction for analysis, and most importantly, the chemical nature of the pigment responsible for the characteristic blue color.

## 2 Results

**Figure 1:**
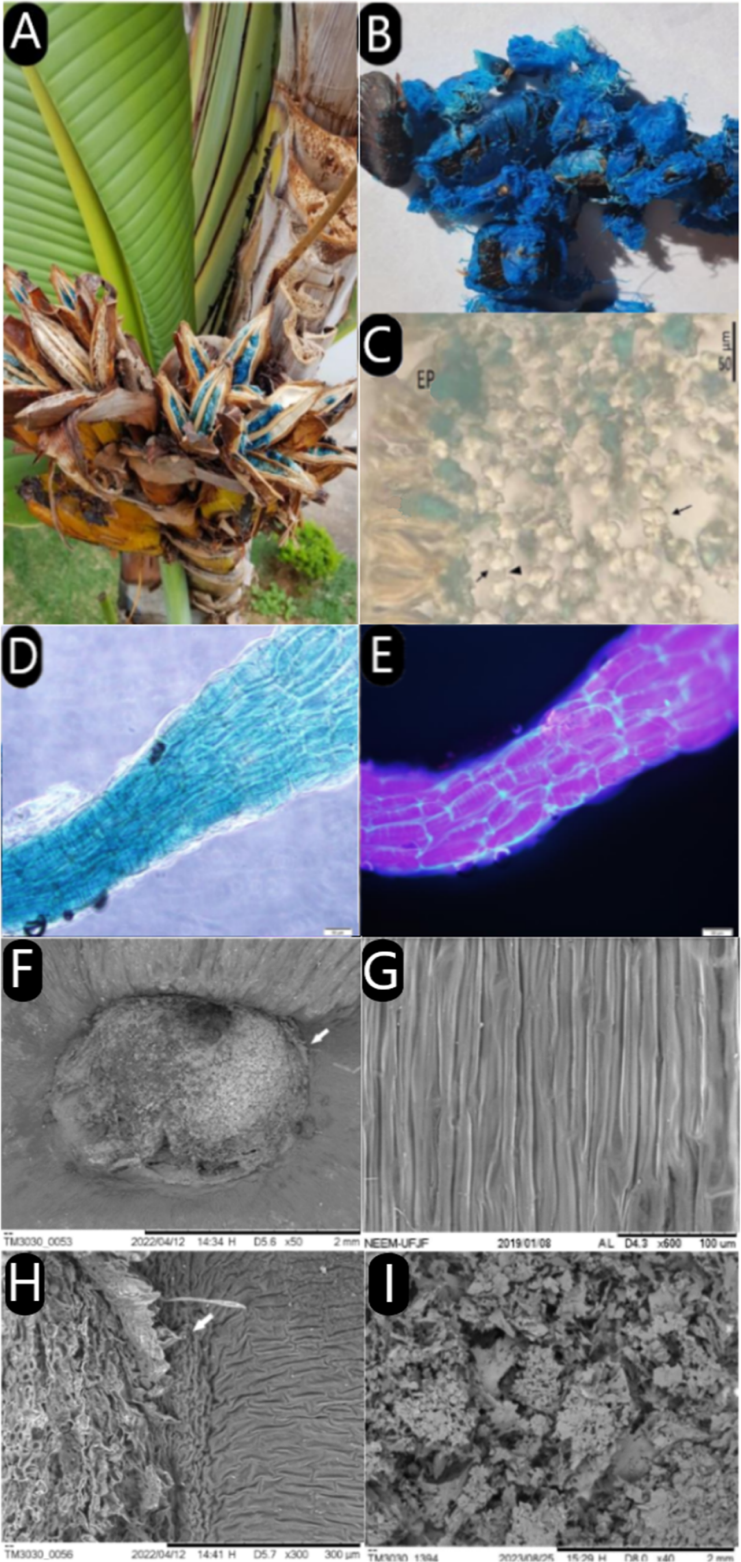
Photographs of the tree and seeds surrounded by blue arils, histological evaluation of cross sections in brightfield and fluorescence emission evaluation, scanning electron microscopy aspects and lyophilized extract from the blue aril fibers of *Ravenala madagascariensis.* Legend: tree and seeds, histological analysis and cross sections in brightfield and fluorescence of *R. madagascariensis* Arils. The image depicts the tree (A), seeds with blue arils (B), undyed cross sections with empty cells (C), with EP: epidermis, Arrows: thickened parenchymal cell walls, and Arrowhead: thin parenchyma cell walls. Blue aril fiber under bright-field (D) and fluorescence microscopy with DAPI filter (E). SEM analysis of *R. madagascariensis* arils, emphasizing the central seed part with emerging cellular structures (F,H), aril insertion into the seed (H), and aril cells with homogeneously distributed compartments (G). Lyophilized extract in 10% SDS (I).

The persistent color and beauty of *R. madagascariensis* make it a widely cultivated ornamental species around the world (Figure 1A). The elongated fibers that attach to the seed are strikingly vibrant arils that provide a unique aesthetic appeal (Figure 1B). These fibers are structurally robust, contributing to the plant’s ornamental value.

Upon detailed examination, the arils exhibit intricate morphological characteristics under microscopy, showcasing their specialized cellular architecture (Figure 1C–E). This structural complexity, coupled with their vivid pigmentation, underscores their significance in both botanical studies and biotechnological applications. Through energy-dispersive X-ray spectroscopy (EDS) in conjunction with scanning electron microscopy (SEM) (Figure 1F–I) was possible to analyze the elemental composition of the sample. From the visualization of untreated aril samples, the composition was determined as 86% carbon, 11% oxygen, and 3% silicon in the sample. In contrast, the lyophilized extract showed a composition of 52% carbon, 18% oxygen, 3% sodium, 11% aluminum, 7% sulfur, and 6% nitrogen.

### 2.1 Phytochemical Analysis of Aril Extracts

After the drying process, the masses of the extracts of *R. madagascariensis* were quantified and expressed as percentages relative to the initial sample mass used in the extraction. The SDS extract had the highest yield, and all extracts presented a waxy appearance. The phytochemical analysis revealed the presence of alkaloids in all evaluated samples. However, the presence of other metabolites was not observed. Initially, the assessment of coumarins had tested positive in the SDS and ethanol extracts. However, due to the fluorescent characteristic of the sample, the result was considered indeterminate for the presence of coumarins (Table 1).

**Table 1:** Results of the Phytochemical Screening of Different Extracts of *R. madagascariensis*^a^

metabolites	RH	RE	RSDS
aspect&weight	light blue and waxy (48.6%)	yellow-green and waxy (20.4%)	dark green and waxy (84.5%)
alkaloids	+	+	+
triterpenes and steroids	–	–	–
coumarins	ID	ID	ID
phenolic compounds	–	–	–
flavonoids	–	–	–
anthraquinones	–	–	–
saponins	–	–	–
tannins	–	–	–

a (+) Positive; (−) negative; (ID) indeterminate; RH: hexane extract of *R. madagascariensis*; RE: ethanol extract of *R. madagascariensis*; RSDS: 10% SDS extract of *R. madagascariensis*.

### 2.2 Macronutrient Composition

The centesimal analyses of moisture, lipids, proteins, ash content, and carbohydrates conducted in triplicates yielded the results described in Table 2.

**Table 2:** Results of Macronutrients Analysis, Flavonoid and Phenolic Compounds^a^

analyzed compounds	moisture (%)	ash (%)	lipids (%)	proteins (%)	carbohydrates (%)	flavonoid content (mg/g)	phenolic compounds (mg/g)
mean & SD	3.71 ± 0.086	0.78 ± 0.021	81.45 ± 0.008	7.1 ± 0.286	7.03	ND	ND

a Mean; the average percentage value of the three samples. ND: not detectable. SD; standard deviation, a measure of the amount of variation or dispersion in the samples.

### 2.3 Ravenala Lipids Detection by Derivatization and GC/MS Analysis

The high concentration of oils and waxes could be perceived upon manual separation of the arils from the seeds. Preliminary solvent studies showed that organic solvents removed significant quantities of a waxy substance without altering or extracting appreciable amounts of the blue pigment. We subjected a hexane extract of the arils to acid catalyzed transesterification with methanol to obtain the corresponding fatty acid methyl esters of the lipids present. We determined that the main fatty acid components are as follows: 41% palmitic acid (C16:0), 14% stearic acid (C18:0), 34% oleic acid (C18:1 ω9), predominantly in its trans isomer form (elaidic acid), and 7% linoleic acid (C18:2 ω6). These do not represent the total composition, as other minor components may also be present. The relatively high percentage of unsaturated fatty acids explains the semisolid nature of the ariĺs lipid fraction.

### 2.4 Solubility Tests

Preliminary solubility tests upon the arils as described in the Experimental Section. Neither heat nor ultrasonication was able to extract the pigment from the arils to which it remained tenaciously bound (no blue color observed in solution). The following solvents were examined: Ethyl ether, acetone, tetrahydrofuran, chloroform, ethyl acetate, ethanol, nitromethane, methanol, dimethylformamide, dimethyl sulfoxide, water, glycerine, dimethylacetamide, 10% trichloroacetic acid, acetic acid, formic acid, 10% aqueous ascorbic acid, 10% FeSO_4_ and last, 10% NaHSO_3_. The solvent at times would acquire a blue coloration but this was due to suspended arils which were removed by filtration. The only solvent capable of solubilizing the chromophore was warm acetic acid. It was noted that after 2 weeks at room temperature and ambient light the solution degraded with formation of a red-brown precipitate. A 10% solution of sodium dodecyl sulfate was also able to extract the blue pigment and showed considerably better stability.

### 2.5 Factorial Design for the Extraction of the Blue Pigment

For the selection of the optimal extraction, specific pH ranges and wavelengths were defined during the factorial design. Optical density (OD), which became the metric for the response pattern in the factorial design, was chosen based on the scanning of samples in a spectrophotometer and the identification of the maximum absorption OD of the extract, found around 620 nm. On the evaluation, a 3^2^ factorial design (3 levels and 2 variables) was used, the time factor was fixed at 24 h, and the variations in the extractant concentration and pH increased from two to three, with a lower, intermediate, and higher value. The extractant concentration varied at 9%, 10%, and 11%, and the pH varied at 5, 5.5, and 6. The results were evaluated, and it was concluded that the best extraction method was tested in extract number 4, with a pH of 5 and the SDS extractant at 10% (Table 3).

**Table 3:** Results of the 3^2^ Factorial Modeling Design for Optimization of Blue Pigment Extraction Conditions from *R. madagascariensis* Arils^a^

sample	pH	SDS (%)	response (OD 620 nm)
1	5	9	2.0089
2	5.5	9	2.035
3	6	9	1.9564
4	5	10	2.385
5	5.5	10	1.9711
6	6	10	2.1004
7	5	11	1.6976
8	5.5	11	1.5223
9	6	11	1.8587
10	5.5	10	1.9422
11	5.5	10	1.8519

a The samples are organized in ascending numerical order for ease of interpretation, but the preparation order did not follow the representation shown.

**Figure 2:**
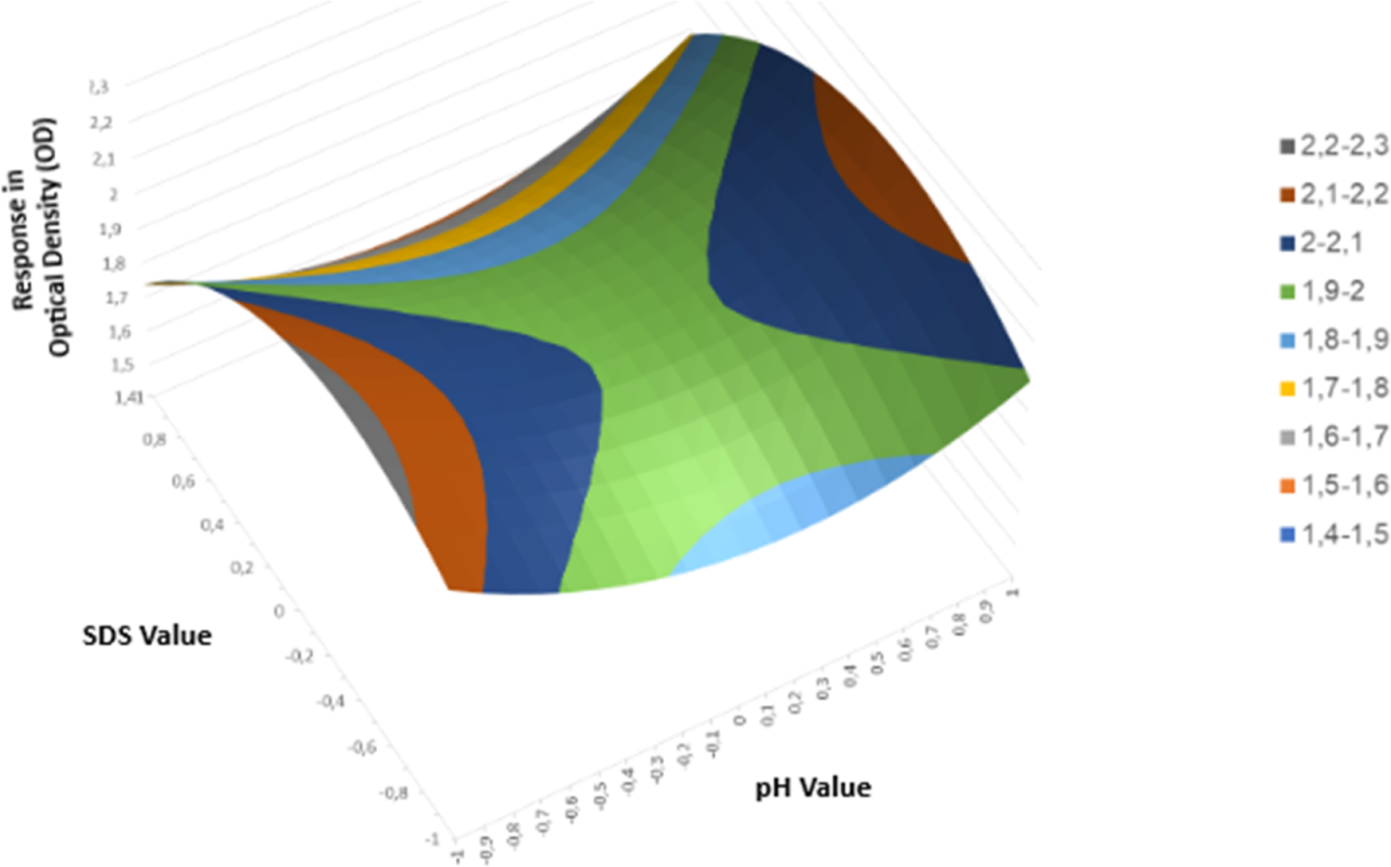
Response surface obtained through the factorial design. Legend: the colors indicate the levels of optical density response on the surface of the assay. Statistical analyses indicated that there is no lack of fit in the experiment.

According to the results obtained and the statistical analyses performed from the triplicates of the central point of the assay, with a variance of 0.003 and a relative standard deviation in terms of percentage equal to 0.2%, it was possible to conclude that variations in pH are not significantly impactful for the best optical density response regarding the SDS extractant concentration. This conclusion can be observed in Figure 2, where the axes represent the pH and SDS values along with their respective optical density responses.

The standardized extract after the factorial design showed excellent color stability, remaining blue for a considerable period of 52 days of storage at room temperature and natural light exposure.

### 2.6 Protein Characterization by Mass Spectrometry

**Figure 3:**
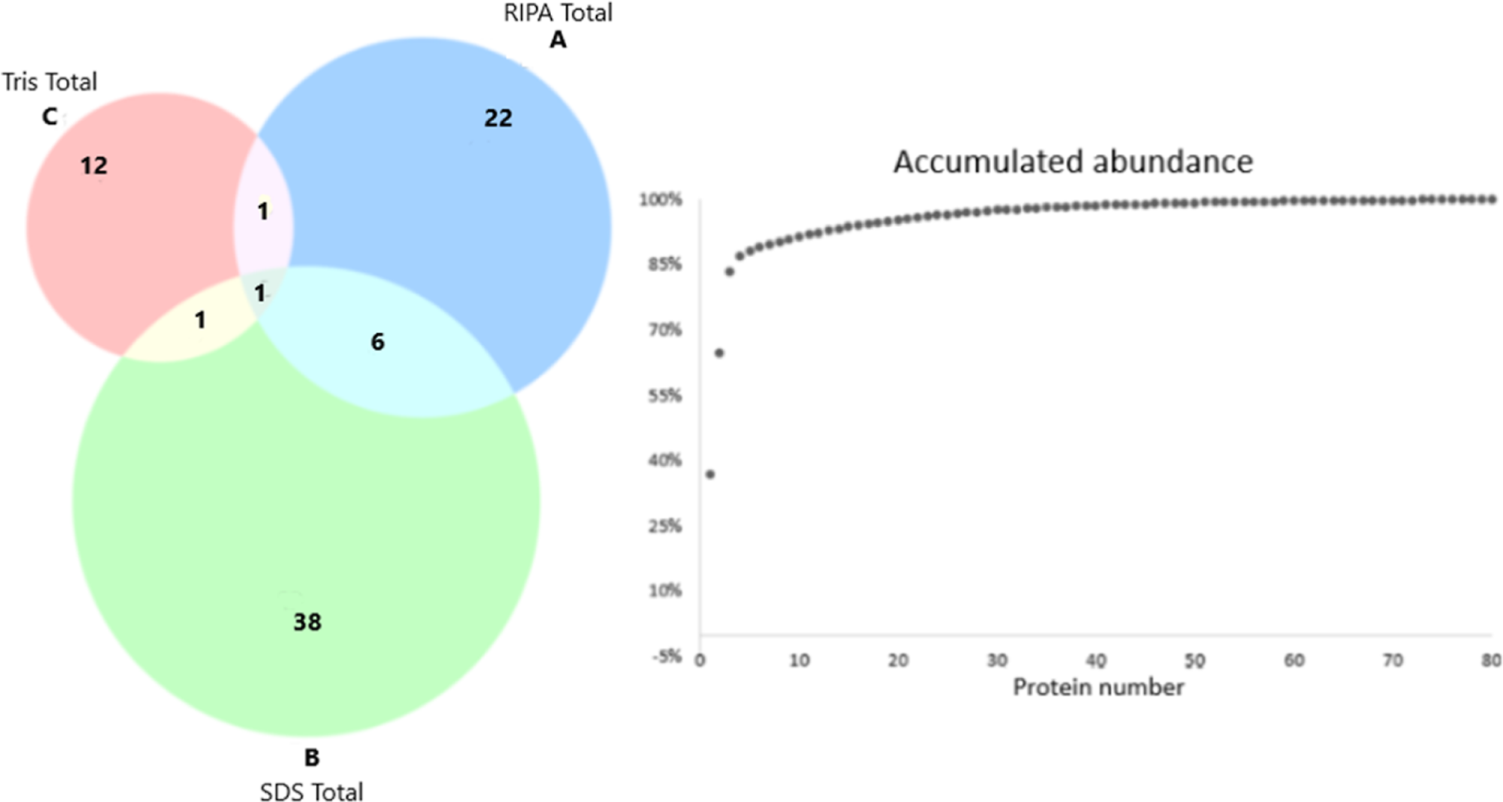
Compositional analysis of the aril proteome of *R. madagascariensis*. Diagram-number of proteins identified for extract solvent: Ripa (A), SDS (B) and Tris (C). Cumulative abundance plot of total proteins Legend: The image depicts a Venn diagram. In (A), proteins identified in the RIPA extract, in (B), proteins from the SDS extract, and in (C), those from the Tris extract.

Until the production of this work, no specific protein database for *R. madagascariensis* was available. However, using a customized database by including protein sequences from the Zingiberales order, through in-gel digestion of both bands from each extract, it was possible to identify 80 nonredundant protein entries (Supporting Information Table). Among these, only 8 were shared between two extraction methods. Six were commonly found by RIPA and SDS extractions, one was detected in both SDS and Tris fractions, and only one was common to all three extraction methods (Figure 3). The SDS-extracted proteins showed better qualitative performance in terms of total proteins. In general, 38 proteins were found exclusively with the SDS method, while RIPA and Tris contributed 22 and 12 proteins, respectively (Figure 3). On the other hand, RIPA extracted proteins were more abundant in area than those from the other methods. Overall, the three most abundant proteins (PPR repeat, Allene oxide synthase, C2 NT-type domain-containing protein) were from RIPA fraction, which together represented approximately 82% of all proteins in this sample (Figure 3—graph).

In this proteomic analysis we found a peptide (SFHNVLEVSK) exhibiting homology to two phytocyanin domain-containing proteins (Uniprot Access Codes: A0A426 × 6F8 and A0A804JMG3). They occupied the 07th and 13th positions in the top 30 most abundant proteins identified. These identities were obtained from one band of the Tris fraction and in both bands of SDS and RIPA extracts (Supporting Information table).

**Figure 4:**
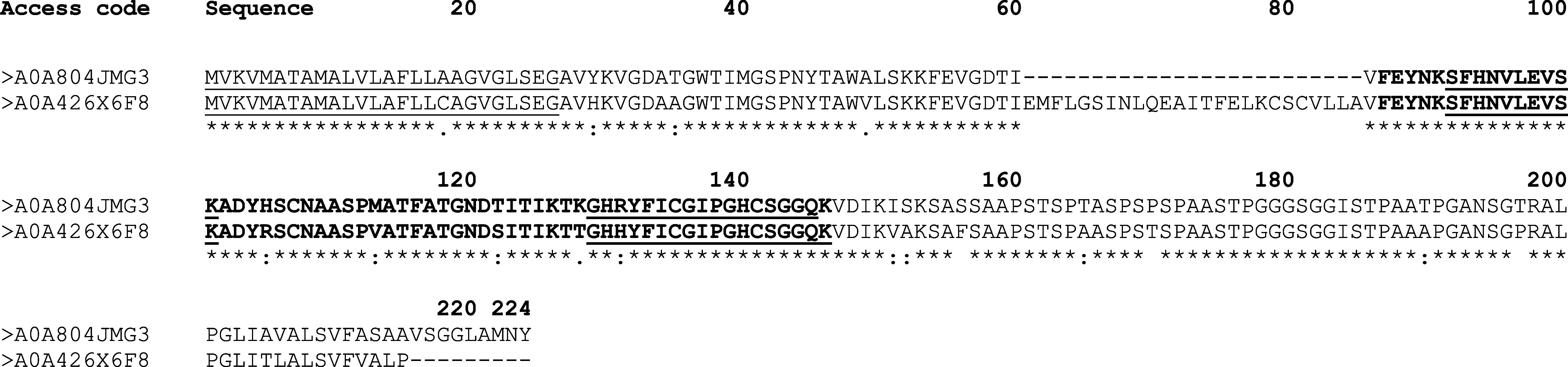
Phytocyanin alignment. Legend: alignment of the peptide sequence from proteomic analysis with Uniprot proteins. Gray highlights the phytocyanin domain, with bold/underlined amino acids marking the blue copper-binding site. N-terminal signal peptides are underlined. Alignment used Clustal Omega, with domains/signals predicted by InterProScan (*) marks identical residues.

Predictions from InterProScan supported that this peptide belongs to a conserved phycocyanin domain. Moreover, this peptide in addition to the two homologous proteins were interrogated against the entire NCBI database, and the most significant hits corresponded to phytocyanins (Figure 4).

Furthermore, using phytocyanin domain as a keyword search in the Zingiberales database, we have obtained 303 results. These findings reinforce that phytocyanins are members of the Zingiberales order. Although our findings support the evidence that the aril proteome contains copper containing proteins, it is important to highlight that a definite proof and the full characterization of blue proteins from *R. madagascariensis* will only be possible when a species specific database is available.

## 3 Discussion

The structures that cover the seeds of some species of the Strelitziaceae family, known as arils, are indeed very interesting. Their often vibrant coloration is eye-catching and has great potential for use as a natural dye, especially the blue arils of the species *R. madagascariensis*, which have a color that is difficult to achieve in nature. Worldwide patents already address the use of arils in cosmetic formulations. In 2017, Andre and Garcia (Dyestuffs of plant origin and use thereof for coloring compositions, in particular cosmetic compositions United States Patent 9839603)^12^ developed a patent related to the use of *R. madagascariensis* arils as natural dyes in waxy, solid, and pasty cosmetic compounds, mainly in makeup but also for use in other body and food products. Later, Manoel and cools, 2019^13^ has also applied the extract of *R. madagascariensis* arils as a dye in stable cosmetic formulations, concluding that the extract is a promising agent for incorporation into pharmaceutical formulations. Although these patents are not very recent, the chemical origin of the active principles used by them, the arils, is not well-known. Understanding this origin, the most appropriate extraction procedure, the biological and physicochemical properties, and incompatibilities are essential for the production of a safe product that meets the expectations targeted with its development.

The literature is still very scarce in descriptions of the histological analysis of *R. madagascariensis* arils. The information available in the literature describes arils as fibrous structures often colored that coat the seeds and are usually associated with animal pollination attracted by the colors of the arils.^10^ For the species *Strelitzia nicolai*, which shares some similarities, the literature provides detailed descriptions of the structures by various methods, as well as the description of the formation and detection of bilirubin in these arils.^14^

In our study, microscopy techniques were used to elucidate the framework of the arils, their morphology, histology, and chemical composition. With the results obtained through optical microscopy analysis, it was possible to observe elongated cells with thick cell walls in the epidermis and a yellowish color. With further cuts, it was also possible to visualize the arrangement of the blue color in the aril. The color is dispersed and inside almost all cells, becoming more prominent toward the interior of the aril fibers. Fluorescence microscopy was also used during the investigation, through which a very curious characteristic of these structures was observed: their fluorescent capacity. When taking a fresh cut of some aril fibers to the microscope, fluorescence was observed throughout the fiber, leading to the conclusion that all that blue pigment internalized in the aril cells is fluorescent. This feature generates interest as a potential value-added in the use of aril extracts as pigments that, in addition to their natural color, would also be capable of bringing fluorescence to the pigmented product.

Scanning electron microscopy revealed the firm and layered appearance of *Ravenala madagascariensis* aril fibers as they attach to the seed. Additionally, this technique provided insights into the chemical composition of the arils. In their natural state, arils primarily consist of a high percentage of carbon with smaller percentages of oxygen and silicon. However, the substantial presence of wax in the arils might have obscured the composition results.

Natural dyes are predominantly derived from plants, encompassing various plant parts such as roots, stems, leaves, fruits, and flowers, producing colors like red, black, brown, yellow, green, and blue.^15^ Secondary metabolites in plants, associated with a range of pigments, contribute to this diverse color palette.^16^ The hues exhibited by *Sterilitziaceae* arils (orange and blue) may be linked to the presence of anthocyanins, glycosylated polyphenolic metabolites distributed in various plant species, belonging to the flavonoid class, and capable of imparting colors such as blue and purple to plants.^17,18^

Given the previously reported data in the literature, our interest was to assess the presence of secondary metabolites in this sample. After conducting classical assays for major classes of secondary metabolites, such as alkaloids, triterpenes, steroids, coumarins, phenolic compounds, flavonoids, anthraquinones, saponins, and tannins, as well as the analysis of flavonoid and phenol content, no positive results were observed that would guide us toward a specific chemical group for further analysis. The positive result for alkaloids might actually be a cross-reaction due to the presence of proteins in the sample, as one of the limitations of the techniques used for the identification of these metabolites is the false-positive result with proteins, purines, alpha-pyrones, some coumarins, hydroxy phenols, and lignans.^19^ The indeterminate presence of coumarins was clarified through the results obtained from the chromatoplate analyses. In this study, phytochemical analysis revealed no correlation between the blue pigment in the arils of *R. madagascariensis* and secondary metabolites. Anthocyanins, which are typically responsible for blue coloration in plants, are known for their instability due to factors such as their chemical structure, the presence of oxygen, pH fluctuations, temperature, and exposure to light. Given the absence of phenols in the samples and the stability of the pigment over a testing period of approximately two months, these findings suggest that anthocyanins do not contribute to the blue color observed in the arils of *R. madagascariensis.*

With the absence of secondary metabolites related to the pigmentation of the arils and results strongly suggesting the presence of proteins in them, centesimal analyses were developed to identify carbohydrate, fat, moisture, and protein content. The results demonstrated carbohydrate and protein quantities close to 7% of the sample, while lipids held a significant percentage of approximately 81%. The high lipid concentration reinforces the barrier that the waxy composition offers for accessing the interior of the arils and for pigment extraction, making SDS the most effective detergent for overcoming this resistance.

The factorial design is a multivariate system for experimental optimization, allowing the evaluation of the effect of a larger number of variables simultaneously, based on a reduced number of experimental assays.^20^ In this work, the design was developed to identify the most appropriate extraction route for the blue pigments in the arils, resulting in a standardization of conditions that improved the pigment extraction. Through this system, we concluded that the variable that most interferes with pigment extraction is the extractor concentration, and the wavelength with the highest absorption peak is in the region of 620 nm.

The chemical nature of the blue pigments in the arils has not been described in the literature. In the development of their patent in 2017, Andre and Garcia^12^ link the color formation in the arils of *R. madagascariensis* to an optical phenomenon known as iridescence. This physically occurring phenomenon would be caused by a region of the plant’s epidermal surface cells, present in a multilayered structure responsible for the light interference mechanism. However, the characteristics demonstrated in our studies lead to the hypothesis that the observed coloration may be related to the presence of chromoproteins. Chromophoric proteins have a connection with chromophores, organic functional groups that absorb in the ultraviolet or visible light region, of a nonprotein nature, responsible for color development.^21^

As mentioned before, in our experiment, the visible spectrum of the SDS extract shows the wavelength with the highest absorption peak at 620 nm, a value within the absorption range where the absorbed color is yellow, and the complementary color is blue-green (595–650 nm), indicating that the color in the arils occurs through the described chemical process. We also observed an absorption peak at 400 nm in our analyses, which may be associated with the partial degradation of the extracted proteins. Pirone and colleagues in 2009 and 2010^22^ identified the presence of bilirubin, a yellow-orange tetrapyrrole produced from the degradation of the heme group by mammals and some other vertebrates, in eight species of angiosperm orders. The authors believe that chromophore groups play an important role in the color composition of the arils of the Strelitziaceae family.

In mammals, the connection between bilirubin and proteins, for example, creates new patterns of light absorption and fluorescence emission for this tetrapyrrole,^23^ similar to those observed in the standardized aril extracts in our study. Bright blue water-soluble pigments have been described in the literature (phycocyanin and allophycocyanin) and are valuable products with various commercial applications, used as natural cosmetic dyes or as fluorescent probes in flow cytometry and immunoassays.^24^

The protein characterization of the blue pigment by electrophoresis indicated the molecular weight of the investigated protein as 14,03 kDa, a reproducible result in various developed assays. Fragments from the gel analyzed by liquid chromatography coupled with high-resolution mass spectrometry led to the identification of phytocyanin domain-containing protein with a precise mass/charge ratio (*m*/*z*), matching known spectra for phytocyanin. Validation of the spectrometry results through additional techniques such as Western blotting could further support the presence of this protein by providing additional confirmation of its size and abundance, thereby strengthening the findings from mass spectrometry. A review of the scientific literature reveals that phytocyanins have been identified in other plants, particularly those with similar pigmentation, underscoring the relevance of these findings. Previous research on the biochemical composition of *R. madagascariensis* has been limited, and this study fills a significant gap by highlighting the role of phytocyanins on it is arils.

## 4 Conclusion

In this work, a characterization and analysis of the blue pigment present in the arils of *R. madagascariensis* were proposed. Morpho-histological analyses using various microscopy techniques revealed the presence of the blue pigment dispersed in the cells of the aril fibers, with a higher concentration of coloration in the interior part. Phytochemical evaluation did not identify any secondary metabolite responsible for the observed blue color in the arils. The factorial planning of blue pigment extraction was able to indicate the optimal extraction conditions, which remained stable for a period close to 2 months at room temperature. The protein characterization of the blue pigment by electrophoresis associated with mass spectrometry suggested that the molecule in question is a phytocyanin domain-containing protein. This significant finding validated by additional methods, could confirm the presence of this protein, contributing to the understanding of plant pigmentation and protein function. This discovery not only elucidates the unique biochemistry of the Traveler’s Palm but also adds to the broader knowledge of phytocyanin roles in plants.

## 5 Experimental Section

### 5.1 Sample Collection and Microscopy Analysis

Blue arils from *R. madagascariensis* plants in Juiz de Fora, MG, Brazil, were collected, manually separated from seeds, and stored at room temperature. A sample was submitted to the Herbário Leopoldo Krieger, Universidade Federal de Juiz de Fora, with registration CESJ 63816, identified by Dr. Fátima Regina Gonçalves Salimena. For morphological evaluation, dry arils were rehydrated in a solution of distilled water, 96% ethyl alcohol, glycerin, and detergent (5:4:1 ratio) for 72 h, then transversely cut using a Ranvier microtome, mounted on glass slides, and covered with coverslips. Fluorescence microscopy involved observing cut fibers on slides using a BX 51 microscope with a DAPI filter and a PowerShot A640 Cannon camera.

Scanning electron microscopy (SEM) analyzed the morphology and chemical composition of blue arils. Fibers from arils attached to seeds and lyophilized extracts (0.1 g arils in 5 mL of 10% SDS solution) were examined. Seeds were directly observed without preparation. Analyses were performed using a Hitachi TM3030 benchtop SEM with the SwiftED3000 EDS module, operating at 15 and 5 kV at the CentralBio—Laboratório Multiusuário de Bioprodutos e Bioprocessos of Faculdade de Farmácia da Universidade Federal de Juiz de Fora.

### 5.2 Phytochemical Analysis

1 g of fresh *R. madagascariensis* arils was added to each of 3 Falcon tubes containing 10 mL of different solvents (hexane, ethanol, or 10% SDS). The extraction process was carried out by static maceration. The arils were left in the solvent for 24 h, then centrifuged at 2000 rpm for 5 min, and the supernatant was removed and stored in an identified tared beaker. This process was repeated three times for hexane and ethanolic extracts. After the process, the extracts were dried to obtain the yield and stored, protected from light and refrigerated. The 10% SDS extracts went through the same process as the organic extracts; however, there was no repetition. Static maceration was performed with 10 mL of solvent for 24 h, then the material was centrifuged at 2000 rpm for 5 min. The supernatant was collected into an identified tared beaker, and drying occurred at room temperature. Preliminary phytochemical analysis, for determining the main chemical classes of secondary metabolites, followed the protocol described by Matos (1997),^25^ with modifications. Solutions were prepared at a concentration of 10 mg/mL (40 mg of each sample in 4 mL of MeOH) for the identification of alkaloids by the precipitation method with Dragendorff, Hager, and Mayer reagents; triterpenes and steroids using the Liebermann–Burchard method; coumarins using a 10% KOH solution; phenolic compounds with a 3% FeCl_3_ solution; flavonoids with a 5% AlCl_3_ solution; anthraquinones through a 0.5 M NaOH solution; saponins through the foam index, and tannins using a gelatin solution. The assays were performed in duplicates.

### 5.3 Identification of Alkaloids, Triterpenes, Steroids, Coumarins, Phenolic Compounds, Flavonoids, and Anthraquinones

Alkaloid identification using Hager, Mayer, and Dragendorff reagents. Positive results were indicated by the presence of white precipitate or turbidity (Hager and Mayer) and an orange color (Dragendorff). Triterpenes and steroids were assessed in another microplate, with positive results marked by blue-green (steroids) and red (triterpenoids) colors. Coumarins were detected by dripping the sample onto filter paper and observing blue fluorescence under UV light. Phenolic compounds were identified by dripping the sample onto filter paper, followed by a 2% FeCl_3_ solution, resulting in a dark blue stain. Flavonoids were detected by observing yellow fluorescence on a filter paper strip under UV light. Anthraquinones were identified in a microplate, with a positive result marked by a red color in the solution.

### 5.4 Determination of Flavonoid Content

The flavonoid content was determined using the method described by Miliauskas et al. (2004)^26^ with some modifications, using rutin as a standard. For the assay of the extracts, a stock solution was prepared at 500 μg/mL in ethanol for organic extracts, and a 1:10 dilution in ethanol was made for SDS 10% extracts. The assays were performed in triplicate and after 40 min of incubation at 20 °C and protected from light, the absorbances of the solutions were recorded at 415 nm in a spectrophotometer. The total flavonoid content was expressed in mg/g of plant extract, in rutin equivalents (RE).

### 5.5 Determination of Phenolic Compounds

The determination of phenol content was performed using visible region spectroscopy with the Folin–Ciocalteu method with modifications,^27,28^ employing tannic acid as a standard. After 30 min of incubation protected from light, absorbance was measured at 770 nm in a spectrophotometer. The assays were performed in triplicate, and the mean ± standard deviation was used to calculate the phenol content. The total phenol content was expressed in mg/g of plant extract, in tannic acid equivalents (TAE).

### 5.6 Macromolecular Composition

The determination of moisture involved atmospheric pressure drying, where approximately 0.5 g of the sample was weighed on a Petri dish and dried in an oven at 105 °C along with purified sand for 2 h. The moisture content was calculated based on the mass loss during drying. To determine lipids, the arils underwent Soxhlet extraction, where approximately 2 g of the sample was weighed into a cartridge and extracted with ether for 8 h. The lipid content was calculated based on the extracted mass. Protein analysis was performed using the Kjeldahl method, involving digestion of the arils in sulfuric acid with catalysts, followed by distillation and titration. The total nitrogen content was used to calculate the protein content. Ash quantification involved incinerating the samples at 550 °C in a preweighed capsule. The ash content was calculated based on the mass difference before and after incineration. Finally, carbohydrate content was determined by difference, subtracting the contents of moisture, lipids, proteins, and ashes. The physicochemical analyses provided essential information about the composition of *R. madagascariensis* arils, contributing to the nutritional characterization of these plant components. These analyses were conducted at the Laboratório de Composição e Valor Nutricional dos Alimentos, located at Departamento de Nutrição da Universidade Federal de Juiz de Fora.

### 5.7 Solubility Tests

A small portion of aril (10 mg) was placed in a test tube with approximately 2 mL of solvent. The aspect of the aril and solvent were observed with manual agitation, ultrasound irradiation and heating in a boiling water bath. The mixtures were filtered through an 0.45 μM PTFE membrane to remove suspended particles before visible observation.

### 5.8 Sample Derivatization and GC/MS Analysis

The waxy solid present in the arils was first extracted with hexane, and the solvent was then evaporated. The resulting residue (50 mg) was saponified with 0.5 M KOH in methanol (MeOH) at 100 °C for 10 min. After cooling, 5 mL of a solution composed of 2 g NH_4_Cl, 60 mL MeOH, and 3 mL H_2_SO_4_ was added, followed by an additional 10 min heating period. A brine solution was introduced, and the mixture was extracted twice with distilled hexane. The solvent was removed, and the oily residue was diluted in hexane (1 mg/mL) for GC/MS analysis.^29^

The GC/MS measurements were performed on a Shimadzu QP2010plus system equipped with a RESTEK Rtx-5 ms (5% diphenyl) column, using helium as the carrier gas. The operating conditions included a split ratio of 1:10, an injection volume of 1 μL, a total flow of 19.1 mL/min, and an injector temperature of 245 °C. The oven temperature program was set as follows: 80 °C (3 min), then 5 °C/min up to 150 °C (hold 6 min), then 5 °C/min to 230 °C (hold 5 min), and finally 15 °C/min to 300 °C (hold 5 min).

### 5.9 Factorial Design for Blue Pigment Extraction

To determine the most appropriate extraction method for the samples, a factorial design was developed, a 3^2^ factorial design, evaluating two factors: pH variation and SDS concentration variation, at three levels: lower (−), intermediate (0), and upper (+). The time was fixed at 24 h as it showed no statistical significance in its variations. Optical density was then assessed at pH 8, 8.5, and 9, with the extractor concentration varying between 9%, 10%, and 11%. In the factorial design, central point repetitions served as model replicates, and statistical calculations of mean, variance, and standard error were performed based on these replicates. The samples from the assay were prepared in a randomized manner, following a predetermined order, but the table is following numerical order for better comprehension. The evaluation was conducted by measuring the emission spectrum of the blue color with a scan in a spectrophotometer (Spectramax 190, Molecular Device) between 400 and 750 nm and can be seen in Table 4.

**Table 4:** 3^2^ Factorial Modeling Design for Optimizing Conditions for the Extraction of Blue Pigment from the Arils of *R. madagascariensis*

sample	TIME (h)	SDS (%)	pH
1	24	9	5
2	24	9	5.5
3	24	9	6
4	24	10	5
5	24	10	5.5
6	24	10	6
7	24	11	5
8	24	11	5.5
9	24	11	6
10	24	10	5.5
11	24	10	5.5

### 5.10 Thin Layer Chromatography of the Acid Digest of Ravenala Arils

Approximately 100 mg of dried, defatted arils were heated in 6 M HCl (2.5 mL) at 80 °C for 15 min. Bovine Serum Albumin (BSA) was used as a positive control. The supernatants were spotted on a Whatman cellulose coated TLC plate and eluted with *n*-butanol/water/acetic acid 3:1:1 followed by revelation with ninhydrin reagent.

### 5.11 Protein Characterization of the Blue Pigment by Electrophoresis

The protein content of the *R. madagascariensis* extracts was assessed through denaturing gel electrophoresis. The standardized extract of the arils in 10% SDS was initially precipitated with trichloroacetic acid (TCA). After centrifugation and removal of the supernatant, the pellet was washed and resuspended in a sample buffer for electrophoresis. All electrophoresis were carried out in a horizontal system using an Amersham ECL Box GE Healthcare support. Initially, a ready-made gel of the same brand was used with an SDS concentration of 8–16%. The gel underwent a prerun of 12 min at 160 V before applying the samples, immersed in Amersham ECL Gel 10× 1:10 running buffer in distilled water, following the manufacturer’s instructions. Subsequently, the samples and the Novex Sharp Prestained Protein Standard with a range between 3.5 and 260 kDa were applied to the gel. The electrophoretic run was performed at a voltage of 140 for 60 min. After this time, the gel was submerged in 1 L of a fixing solution composed of ethanol, acetic acid, and distilled water (40:10:50 v/v) for 30 min. Next, the gel was stained with 0.01% Comassie Brilliant Blue R-250 in 25% ethanol (v/v) and 5% acetic acid (v/v) solution for 10 min. The gel was then washed with distilled water to remove excess dye and placed in a bleaching solution composed of 8% acetic acid (v/v), 25% absolute ethanol (v/v), and 67% distilled water (v/v) until the bands were visualized, with agitation at 60 rpm on a shaker. Finally, the gel was transferred to a storage solution, consisting of 87% glycerol and 13% distilled water (v/v), for an additional 30 min. Afterward, two distinct SDS gels were prepared: one with 12% polyacrylamide and the other with 15% polyacrylamide. Both gels included additional extractors—Tris, RIPA, and SDS—each in triplicate. Those were stained with Coomassie G-250, then unstained and stained with silver nitrate. Subsequently, a new SDS-PAGE gel (12%) was prepared. It was stained with Coomassie G-250, followed by excision of low molecular weight bands and digestion with trypsin. For Tris extracts, 5 μg of proteins were applied and 10 μg for SDS and RIPA extracts.

### 5.12 Protein Characterization by Mass Spectrometry

The preliminary and final analysis for identifying proteins present in *R. madagascariensis* aril extract were performed through in-gel digestion and liquid chromatography coupled with mass spectrometry. The gel bands identified in electrophoresis at approximately 14 kDa were excised and subjected to mass spectrometry analysis. Initially, the samples underwent pretreatment and in-gel digestion using the enzyme trypsin. The resulting tryptic peptides were resuspended in a 0.1% trifluoroacetic acid solution (Fluka), and 5 μL were injected using the nano UHPLC UltiMate 3000 system (Dionex) equipped with a Nano-Trap Acclaim PepMap100C18 column (100 μm i.d. × 2 cm, 5 μm, 100 A; Thermo Scientific) and the C18 Column (75 μm × 10 cm, 3 μm, 120 Å, Thermo Scientific).

The peptides were initially trapped using a mobile phase composed of 2% ACN with 0.05% TFA, and after 3 min, they were eluted at a flow rate of 3 μL/min. These eluted peptides underwent chromatographic separation on a C18 column using a mobile phase composed of 0.1% formic acid, 80% ACN, and 0.1% formic acid, with a flow rate of 0.300 μL/min under a gradient mode of 4 to 90% ACN in 61 min, at 40 °C.

The nanoUHPLC system is coupled to the Q-Exactive Thermo Scientific instrument through a Nanospray Flex Ion Thermo Scientific source, conducting mass spectrometry analysis of the eluted peptides. The Nanospray Flex Ion source is equipped with a stainless steel nanobore emitter (150 μm o.d. × 30 μm i.d., Proxeon, Thermo Scientific) and operated at a voltage of 3.45 kV in positive mode with a temperature of 250 °C. A scan was performed at a resolution of 70,000 with a maximum injection time of 120 ms and ion accumulation at a value of 1 × 10^6^. Ion fragmentation occurred in the higher-energy collisional dissociation (HCD) cell, and in this method, the 12 most intense ions were selected for monitoring in the range of 300–2000 *m*/*z*, with charges +2 and +5, isolated within a 2 *m*/*z* range before fragmentation, with a normalized collision energy of 30 V. The generated MS/MS spectra were acquired at a resolution of 17,500, with a maximum injection time of 150 ms and ion accumulation at a value of 5 × 10̂5 ions. The exclusion time used was 40 s. Subsequently, the obtained mass spectrum was subjected to a database search using PEAKS v8.5 software. After peptide identification, a BLAST analysis and alignment with the THU71878.1 sequence of *Musa balbisiana* at Uniprot database for plant proteins from the Zingiberales order, which includes 359,172 sequences downloaded on February 29, 2024. The selection criteria for peptides set by the software included up to two missed cleavages, fixed carbamidomethylation at cysteine residues, and variable oxidation of methionines. Acceptable error rates were defined as up to 0.1 Da for product ions and up to 10 ppm for precursor ions. Additionally, data were filtered and underwent analysis considering those with an average local confidence (ALC) of 80% or higher, and homology searches were conducted using BLAST against sequences available on NCBI, focusing on the search for Zingiberales proteins.

As per the Journal’s purity requirements, we confirm that all compounds utilized are >95% pure by HPLC analysis.

## Supporting Information Available

This manuscript includes Supporting Information containing additional results, experimental details, and methods, as well as supplementary figures and tables to complement the main text (PDF)

## Abbreviations

EDS: energy-dispersive X-ray spectroscopy
SEM: scanning electron microscopy
SDS: sodium dodecyl sulfate
TLC: thin layer chromatography
RIPA: radio-immunoprecipitation assay
GC/MS: gas chromatography/mass spectrometry
RE: rutin equivalents
TAE: tannic acid equivalents
HCD: higher-energy collisional dissociation
ALC: average local confidence
