# Supplementary material for: Regarding the Nature of the Blue Pigment in Arils of Ravenala Madagascariensis Sonn. (Strelitziaceae)
Source: ACS Omega. 2025 Mar 26;10(13):13350–60. doi: 10.1021/acsomega.4c11177 (PMC11983200; doi:10.1021/acsomega.4c11177)
Supplement: Supplementary file 1 — ao4c11177_si_001.pdf [file ao4c11177_si_001.pdf]

REGARDING THE NATURE OF THE BLUE PIGMENT IN ARILS OF *RAVENALA MADAGASCARIENSIS* SONN. (STRELITZIACEAE)

Beatriz Paiva Nogueira[1], Marcos Miguel Quimas do Amaral[1], Arthur Girard Carpanez[1,3], Ari Sergio de Oliveira Lemos[1], Frederico Francisco Fernandes[4], Nádia Sílvia Somavilla[2], Marcone Augusto Leal de Oliveira[3], William de Castro Borges[4], Eveline Gomes Vasconcelos[1], Richard Michael Grazul[3], Priscila de Faria-Pinto[1*]

1 - Departamento de Bioquímica, Instituto de Ciências Biológicas, Universidade Federal de Juiz de Fora, 36036-900, Juiz de Fora, MG, Brazil

2 - Departamento de Botânica, Instituto de Ciências Biológicas, Universidade Federal de Juiz de Fora, 36036-900, Juiz de Fora, MG, Brazil

3 - Departamento de Química, Instituto de Ciências Exatas, Universidade Federal de Juiz de Fora, 36036-900, Juiz de Fora, MG, Brazil

4 - Laboratório de Enzimologia e Proteômica, Universidade Federal de Ouro Preto, 35400-000, Ouro Preto, MG, Brazil

***Corresponding author: Departamento de Bioquímica, ICB, Universidade Federal de Juiz de Fora, Rua José Lourenço Kelmer s/n, Campus Universitário, Bairro São Pedro; 36036-900, Juiz de Fora, MG, Brazil, e-mail: priscila.faria@ufjf.br**

Supporting Information

TABLE OF CONTENTS

The title page…………………………………………………………………………...S1
Factorial design for the extraction of the blue pigment …………………………………S2
Table S1 - Exploratory 2^3 factorial design for optimizing conditions for the extraction of blue pigment from the arils of *R. madagascariensis*………………………………………….…S3
Table S2 - 3^2 factorial modeling design for optimizing conditions for the extraction of blue pigment from the arils of *R. madagascariensis*………………………………………………...S4
Figure S1 - Evaluation of the color stability of the extract from *R. madagascariensis* arils in a 10% SDS solution maintained at room temperature………………………………………S4
Selection of the pH range and wavelength of maximum UV-VIS light absorption for the SDS extract of arils……………………………………………………………………S5
Figure S2 - Selection of pH and determination of the wavelength of maximum light absorption were carried out for the most efficient extraction of pigment from the samples……………..S5
Table S3 - Results of the Exploratory 2^3 Factorial Design for Optimization of Blue Pigment Extraction Conditions from *R. madagascariensis* Arils……………………………………S6
Thin-Layer Chromatography for the Analysis of Phytoconstituents, Peptides, and Antioxidant Capacity……………………………………………………………………S7
Figure S3 - Chromatoplate of ethanolic, hexane, and 10% SDS extracts for the analysis of the presence of coumarins……………………………………………………………………..S7
Figure S4 - Chromatoplates of ethanolic, hexane, and 10% SDS extracts revealed with DPPH for the identification of antioxidant activity…………………………………………………S7
Figure S5 - Chromatoplate of ethanolic, hexane, and 10% SDS extracts revealed with Ninhydrin for the identification of the presence of peptides…………………………………S8
Protein characterization by mass spectrometry…………………………………………..S9
Figure S6 - SDS 10%, RIPA and Tris HCl Extracts…………………...……………………S9
Table S4 - Top 30 most abundant proteins from *R. madagascariensis* aril proteome……...…S9

Factorial design for the extraction of the blue pigment.

To determine the most appropriate extraction method for the samples, a factorial design of experiments was employed. This involved the organization of the experimental design, data compilation, and experiment optimization. Initially, as the response of the samples to the variables was unknown, an exploratory assay was conducted following a 2^3 factorial design, analyzing three factors: time, pH, and SDS concentration, each with two levels, higher (+) and lower (-). In this initial assay, variations in time (24h and 48h), pH values between 5 and 6, and between 8 and 9, and SDS extractor concentration at 2% and 10% were assessed. About 0.1g

of untreated arils, previously treated with hexane, was weighed in Falcon tubes and added to 5 ml of extracting solution (SDS 2% or 10% solubilized in deionized water). The samples were macerated using a Potter tool. The pH was measured using a benchtop pH meter, and pH values were adjusted using TRIS salt for basic regulation or 0.5 M hydrochloric acid for acid adjustment. After pH adjustments, the samples were subjected to an ultrasonic bath for 15 minutes and stored at room temperature for 24h or 48h. Hexane treatment was performed as an attempt to remove waxy components from the arils and improve pigment extraction. One gram of arils was weighed in a beaker, and 15 ml of PA hexane was added. The solution was kept at room temperature until the sample dried, and the process was repeated twice. The samples were prepared in a randomized manner, following the sequence determined by the table below.

Table S1- Exploratory 2^3 factorial design for optimizing conditions for the extraction of blue pigment from the arils of *R. madagascariensis*.

SAMPLE	TIME	SDS	pH
5	24h	2%	5-6
6	48h	2%	5-6
3	24h	10%	5-6
2	48h	10%	5-6
8	24h	2%	8-9
1	48h	2%	8-9
7	24h	10%	8-9
4	48h	10%	8-9

From the results of the exploratory assay, a second assay was developed, a 32 factorial design, evaluating two factors: pH variation and SDS concentration variation, at three levels: lower (-), intermediate (0), and upper (+). The time was fixed at 24 hours as it showed no statistical significance in its variations. Optical density was then assessed at pH 8, 8.5, and 9, with the

extractor concentration varying between 9%, 10%, and 11%. Sample preparation followed the pattern of the exploratory assay. In the factorial design, central point repetitions served as model replicates, and statistical calculations of mean, variance, and standard error were performed based on these replicates. The samples from the second assay were also prepared in a randomized manner, following a predetermined order. The evaluation in both assays was conducted by measuring the emission spectrum of the blue color with a scan in a spectrophotometer (Spectramax 190, Molecular Device) between 400 nm - 750 nm and can be seen in the next table.

Table S2 - 3^2 factorial modeling design for optimizing conditions for the extraction of blue pigment from the arils of *R. madagascariensis.*

SAMPLE	TIME (fixed)	SDS	pH
3	24h	9%	6
6	24h	10%	6
9	24h	11%	6
8	24h	11%	5.5
4	24h	10%	5
11	24h	10%	5.5
10	24h	10%	5.5
1	24h	9%	5
7	24h	11%	5
2	24h	9%	5.5
5	24h	10%	5.5

Figure S1 - Evaluation of the color stability of the extract from *R. madagascariensis* arils in a 10% SDS solution maintained at room temperature.

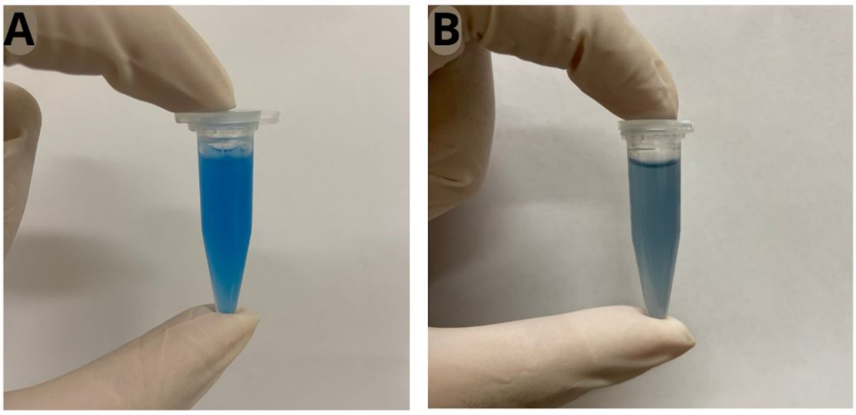

Legend: In (A), the extract of arils in 10% SDS after 24 hours of extraction. In (B), the same extract after 52 days of being kept at room temperature.

Selection of the pH range and wavelength of maximum UV-VIS light absorption for the SDS extract of arils

For the selection of the optimal extraction, specific pH ranges and wavelengths were defined during the factorial design. Optical density (OD), which became the metric for the response pattern in the factorial design, was chosen based on the scanning of samples in a spectrophotometer and the identification of the maximum absorption OD of the extract, found around 620nm. The pH range was evaluated through tests with variations between acidic and basic conditions. The basic extract was adjusted using TRIS salt, and the acidic extract was adjusted with 0.5M HCl. Analyzing the optical densities of these samples resulted in the most suitable pH range for the factorial design.

Figure S2 - Selection of pH and determination of the wavelength of maximum light absorption were carried out for the most efficient extraction of pigment from the samples.

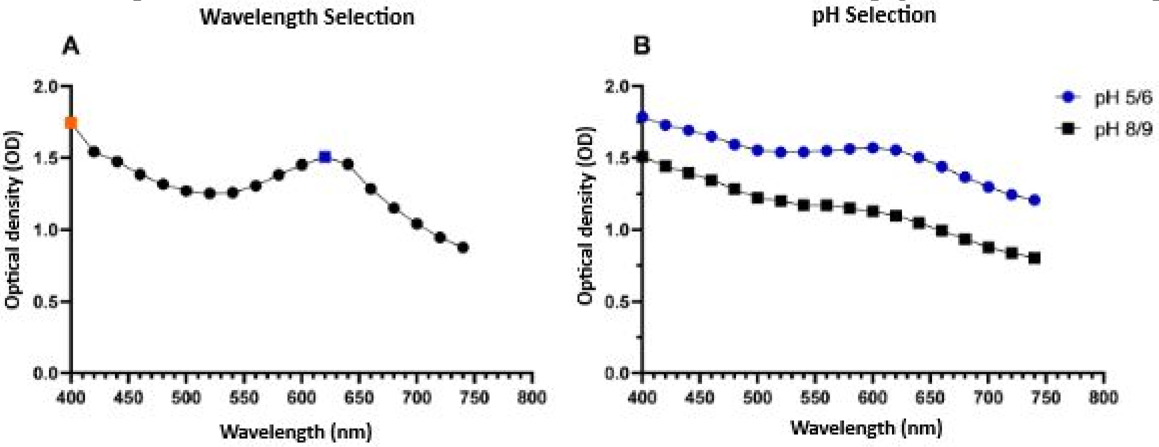

Legend: In (A), the graph represents the visible light spectrum scan of an extract from *R. madagascariensis* to identify the OD of maximum absorption. Highlighted in orange is the peak at 400nm, and in blue is the peak at 620nm. In (B), the evaluation of the pH of two extracts from *R. madagascariensis* to show in which environment, acidic or basic, the best pigment extraction occurs. Highlighted in blue are the extracts with a pH between 5 and 6.

In the exploratory assay, three factors were evaluated, each with two variations, for the extraction: time (24h and 48h), SDS extractant concentration (2% and 10%), and pH (5/6 and 8/9). In addition, there was a repetition with prior treatment with hexane to identify if the removal of the waxy component from the sample would improve the extraction. The results were arranged in optical density values obtained by spectrophotometry.

Table S3 - Results of the Exploratory 2^3 Factorial Design for Optimization of Blue Pigment Extraction Conditions from *R. madagascariensis* Arils.

SAMPLES	TIME	SDS	pH	R1_HEX (OD 620nm)	R2_SHEX (OD 620nm)
1	24h	2%	5-6	0.53895	1.50615
2	48h	2%	5-6	1.0154	1.2565
3	24h	10%	5-6	0.483	1.5546
4	48h	10%	5-6	1.20945	0.86175
5	24h	2%	8-9	1.06075	1.11195
6	48h	2%	8-9	0.9095	0.7387
7	24h	10%	8-9	0.8291	1.0981
8	48h	10%	8-9	0.80905	1.03895

Legend: R1_HEX: Responses from the assay with prior treatment with hexane; R2_SHEX: Responses from the assay without prior treatment with hexane. The yellow highlight indicates

the best treatment. The samples are organized in ascending numerical order for ease of interpretation, but the preparation order follows the representation shown in Table 1.

With the obtained results, it was possible to show that the hexane treatment does not improve the extraction, and the best variations of the evaluated factors are those from sample 3 with extraction at 24 hours, SDS concentration at 10%, and pH value between 5 and 6. In the exploratory factorial design, no significant influence of the time variable was observed. The variation of low and high time levels, while keeping the other variables fixed, did not result in impactful changes in the observed responses. Therefore, to optimize the extraction, the time was fixed at the low level (24 hours), and a new assay exploring the best result obtained in the exploratory design was developed.

Thin-Layer Chromatography for the Analysis of Phytoconstituents, Peptides, and Antioxidant Capacity

In order to identify the classes of chemical constituents present in the samples and the antioxidant capacity of *R. madagascariensis* extracts, thin-layer chromatography (TLC) was performed. The presence of coumarins, peptides, and the antioxidant activity of the sample were evaluated using specific revelators and mobile phases for each. The results did not indicate the presence of coumarins in any of the three evaluated extracts (ethanolic, hexane, and 10% SDS). On the other hand, Ninhydrin revelation showed positive results in the ethanolic and hexane extracts with the appearance of a pink band at Rf=0.6, and negative results in the 10% SDS extract. The analysis of antioxidant activity using 0.01% DPPH revelator showed positive results in the ethanolic and 10% SDS extracts with the appearance of a clear band at Rf near 0.0, indicating antioxidant activity, and negative results for the hexane extract, as no clear bands were observed after revelation.

Figure S3 - Chromatoplate of ethanolic, hexane, and 10% SDS extracts for the analysis of the presence of coumarins.

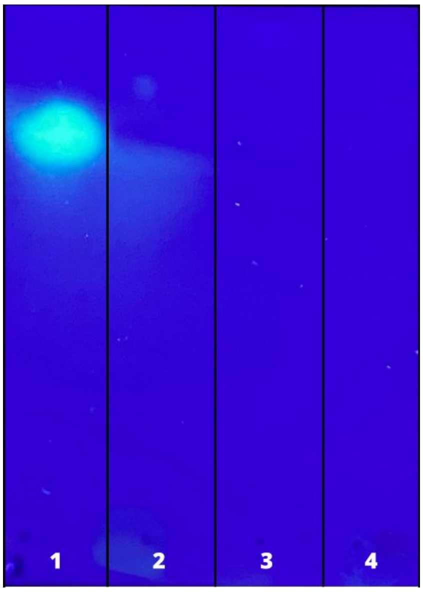

Legend: Chromatoplate for coumarin revelation with (1) used coumarin standard; (2) ethanolic extract; (3) hexane extract; (4) 10% SDS extract. Greenish fluorescence as a marker for positive results.

Figure S4 - Chromatoplates of ethanolic, hexane, and 10% SDS extracts revealed with DPPH for the identification of antioxidant activity.

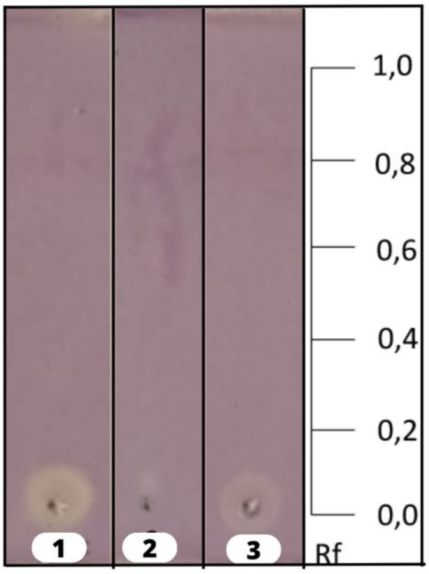

Legend: Chromatoplate of ethanolic extract (1); Hexane extract (2) and 10% SDS extract (3) revealed with DPPH reagent to investigate the antioxidant activity of the extracts.

Figure S5 - Chromatoplate of ethanolic, hexane, and 10% SDS extracts revealed with Ninhydrin for the identification of the presence of peptides.

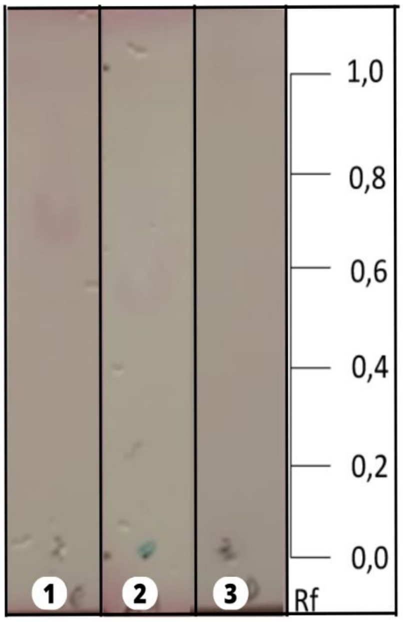

Legend: Chromatoplate of ethanolic extract **(1)**; Hexane extract **(2)** and 10% SDS extract **(3)** revealed with Ninhydrin reagent to investigate the presence of peptides. Pink band as a marker of positivity.

Protein characterization by mass spectrometry

Figure S6 - SDS 10%, RIPA and Tris HCl Extracts

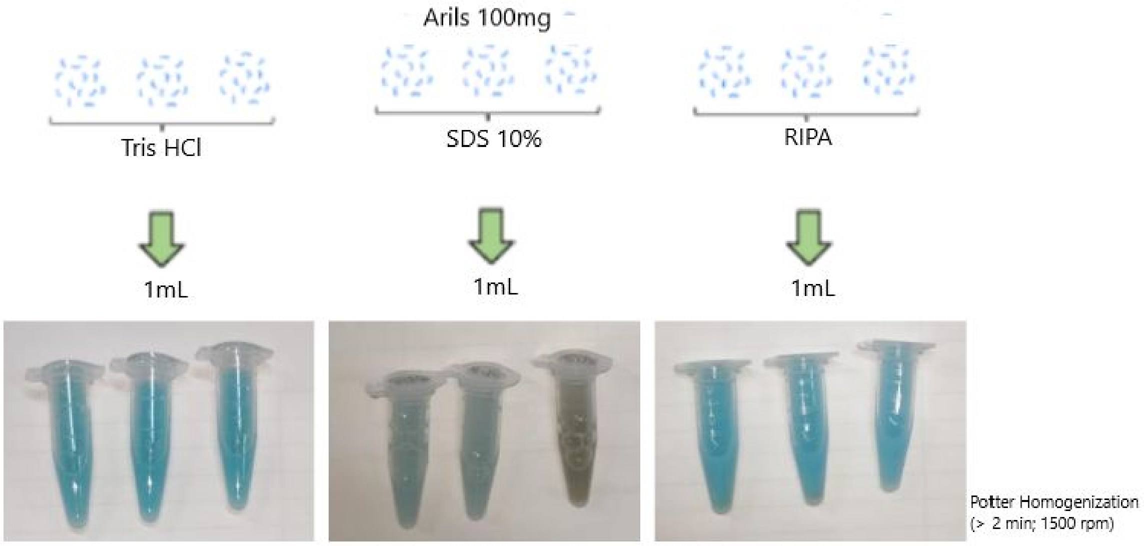

Legend: Preparation of three extract types, each in triplicate, using 10mg of dried arils in 1ml solutions: SDS 10%, RIPA (1% Triton X-100, 1% deoxycholate), and Tris HCl (50 mM).

Table S4: Top 30 most abundant proteins from *R. madagascariensis* aril proteome.

Description	Specie	Cov. (%)	Area	m/z	Accession	Orig.
PPR repeat	*Musa troglodytarum*	2	5.32E+08	45027	A0A9E7KB94	Ripa
Allene oxide synthase	*Zingiber officinale*	2	4.02E+08	53759	A0A8J5KSQ0	Ripa
C2 NT-type domain-containing protein	*Zingiber officinale*	1	2.71E+08	156928	A0A8J5LQD1	Ripa
Uncharacterized protein	*Ensete ventricum*	12	5.01E+07	8046	A0A444FBP7	Tris
DUF3741 domain-containing protein	*Ensete ventricum*	1	1.88E+07	103952	A0A427A7G3	Ripa
BSD domain-containing protein	*Musa balbisiana*	2	1.06E+07	52395	A0A4S8JAY9	Ripa
(wild Malaysian banana) hypothetical protein	*Musa acuminata spp. malaccensis*	5	9.30E+06	19939	A0A804JMG3	Tris
Uncharacterized protein	*Ensete ventricum*	12	8.51E+06	15494	A0A444C5T4	Ripa
Fructose-bisphosphate aldolase	*Ensete ventricum*	3	8.24E+06	49407	A0A444DNW 1	Ripa
Enoyl-[acyl-carrier-protein] reductase (NADH)	*Ensete ventricum*	5	7.89E+06	40473	A0A444CKX3	SDS
Nucleoside diphosphate kinase	*Ensete ventricum*	22	7.28E+06	19639	A0A426ZSC3	SDS
glutathione transferase	*Ensete ventricum*	14	6.73E+06	26592	A0A426Y243	SDS
Phytocyanin domain-containing protein	*Ensete ventricum*	5	6.47E+06	21826	A0A426X6F8	SDS
acetylornithine transaminase	*Musa acuminata spp. malaccensis*	2	6.38E+06	50400	A0A804LAZ8	Ripa
Uncharacterized protein	*Musa troglodytarum*	1	5.66E+06	58394	A0A9E7KXK8	Ripa
Actin	*Ensete ventricum*	2	4.92E+06	51849	A0A426YPF0	Ripa
Glyceraldehyde-3-phosphate dehydrogenase	*Ensete ventricum*	6	4.87E+06	35496	A0A426Y4U8	Ripa
glutathione transferase	*Musa balbisiana*	5	4.81E+06	49572	A0A4S8IJM5	SDS
Protein low PSII accumulation 1 chloroplastic	*Zingiber officinale*	2	4.73E+06	58418	A0A8J5GQ84	Ripa
Histone H4	*Zingiber officinale*	5	4.58E+06	25013	A0A8J5FF06	SDS
Cystatin domain-containing protein	*Ensete ventricum*	11	4.05E+06	21655	A0A445MDU 2	Ripa
VOC domain-containing protein	*Ensete ventricum*	15	3.70E+06	13902	A0A426Y419	SDS
Cystatin domain-containing protein	*Ensete ventricum*	12	3.50E+06	11631	A0A426WXT 3	Ripa
Tubulin alpha chain	*Zingiber officinale*	1	3.49E+06	96545	A0A8J5GF34	Ripa
Nucleotide diphosphate kinase	*Zingiber officinale*	5	3.38E+06	34877	A0A8J5GFW0	SDS
RRM domain-containing protein	*Zingiber officinale*	5	2.97E+06	29461	A0A8J5EV62	Ripa
Histone H4	*Zingiber officinale*	9	2.85E+06	28649	A0A8J5F935	SDS
Actin	*Ensete ventricum*	6	2.83E+06	44771	A0A426XQR4	SDS
Superoxide dismutase [Cu-Zn]	*Zingiber officinale*	16	2.76E+06	15127	A0A8J5GL54	Tris
Uncharacterized protein	*Zingiber officinale*	2	2.18E+06	46311	A0A8J5CE67	Ripa

Legend: The table includes the following details for each protein: description, species of origin, sequence coverage (Cov.%), signal intensity (Area), mass-to-charge ratio (m/z), accession number, and extraction method (RIPA, SDS, or Tris). The three most abundant proteins are highlighted at the top, and the *phytocyanin domain-containing protein* is specifically marked in gray to emphasize its relevance to the study.
